# Body Shame in 7–12-Year-Old Girls and Boys: The Role of Parental Attention to Children’s Appearance

**DOI:** 10.1007/s11199-023-01385-7

**Published:** 2023-06-07

**Authors:** Chiara Pecini, Gian Antonio Di Bernardo, Eleonora Crapolicchio, Loris Vezzali, Luca Andrighetto

**Affiliations:** 1 Department of Education, University of Genoa, Genova, 16128 Italy; 2 Department of Education and Human Sciences, University of Modena and Reggio Emilia, Reggio Emilia, Italy; 3 Department of Psychology, Catholic University of the Sacred Heart of Milan, Milano, Italy; 4 Medical and Dental Department of Morphological Sciences related to Transplant, Oncology and Regenerative Medicine, University of Modena and Reggio Emilia, Modena, Italy

**Keywords:** Body shame, Children, Gender, Parent, Tripartite influence model, Objectification theory, Body image, Parent-child relations

## Abstract

Guided by the Tripartite Influence Model and Objectification Theory, we examined whether parents’ attention to their children’s appearance was related to higher body shame in girls and boys. In Study 1 (*N* = 195) and 2 (*N* = 163), we investigated 7-12-year-old children’s metaperceptions about parents’ attention to their appearance and its association with children’s body shame. In Study 3, we examined the link between parents’ self-reported attention to their children’s appearance and children’s body shame among parent-child triads (*N* = 70). Results demonstrated that both children’s metaperceptions and fathers’ self-reported attention to children’s appearance were associated with body shame in children. Furthermore, when mothers’ and fathers’ attitudes toward their children were analyzed simultaneously, only fathers’ attention to their children’s appearance was associated with greater body shame in girls and boys. Notably, no gender differences emerged, suggesting that parents’ attention to their children’s appearance was not differentially related to body shame in girls and boys. These results remained significant when controlling for other sources of influence, namely peer and media influence, both of which were found to have a strong association with body shame in children. Theoretical and practical implications of our findings are discussed.

Body image concerns are showing a rapid increase in most Western societies and deeply affect younger generations (Shriver et al., 2013). Children starting from three years of age have negative attitudes toward overweight individuals (Spiel et al., 2012) and may develop body image concerns as early as five years old (Davison & Birch, 2002; Davison et al., 2003). During school years (i.e., 6–12 years old), body image concerns become particularly pervasive, and children express them in the same way adolescents and adults do (Ricciardelli et al., 2009). For example, many girls and boys are unhappy with their physical appearance (Hill et al., 1994; Slater & Tiggemann, 2016; Tatangelo & Ricciardelli, 2013) and are worried about how they look (for a review, see Smolak, 2012). Although expressions of body image concerns may be similar across children, adolescents, and adults, the sources for these negative feelings involving body image may differ. Thus, a comprehensive understanding of the correlates of this phenomenon during an early developmental stage is fundamental to prevent and mitigate its consequences.

The present research examined the intergenerational roots of children’s body shame, by specifically focusing on school-aged girls and boys aged 7–12 years. In doing so, we integrated the Tripartite Influence Model (Thompson et al., 1999) and Objectification Theory (Fredrickson & Roberts, 1997). We first analyzed whether children’s metaperceptions about parents’ attention to their appearance would be associated with their levels of body shame, and whether this could differ between genders (Study 1). Next, we analyzed the separate influence of metaperceived paternal and maternal attention to children’s appearance on children’s body shame (Study 2). In Study 3, we validated these results by involving parent-child triads and examining the relationships between mothers’ and fathers’ self-reported attention to children’s appearance and body shame in girls and boys.

## Body Image Concerns: Integrating Two Theoretical Frameworks

Body image concerns refer to experiences of body size misperception and/or negative attitudes or feelings toward one’s own body (Cash & Szymanski, 1995). These concerns manifest in several ways, such as body dissatisfaction, drive for thinness and/or muscularity, poorer body esteem, or body shame, which is the focus of the present work (for a review, see Ricciardelli & McCabe, 2001).

The Tripartite Influence Model (Thompson et al., 1999) represents a sociocultural model of the development of body image concerns that identifies the role of parents, peers, and the media as prominent sociocultural influences on body image. According to the model, these sources of influence promote the internalization of beauty standards and engagement in body comparisons, which negatively affect body image.

Objectification Theory (Fredrickson & Roberts, 1997) proposes that direct and indirect exposure to the sexual objectification of women leads women to adopt a third-person perspective on their own bodies and regularly monitor their appearance. This experience, termed self-objectification, causes poorer psychological well-being, including body shame (for a review, see Roberts et al., 2018).

In the last few years, a number of studies (e.g., Brewster et al., 2019; Frederick et al., 2022; Strübel et al., 2020; Tylka & Andorka, 2012; Velez et al., 2016) tested the integration of these two models to explore the emergence and expression of body image concerns, by considering a variety of samples (e.g., adult cisgenders, adult transgenders), possible sources (e.g., peers, significant others, the media), and multiple psychological mechanisms leading to negative body image, such as the internalization of beauty standards and self-objectification.

Consistent with this emerging literature, we adopted an integrated view of the Tripartite Influence Model (i.e., the type of sources) and Objectification Theory (i.e., the message conveyed by the source) to explore a possible source of body image concerns, i.e., parents’ attention to their children’s appearance, within a relatively neglected age group (i.e., girls and boys aged 7–12 years). Across our studies, we investigated the influence of parents on children’s body image concerns and verified its effects when considered together with those of peers and the media. Specifically, by adapting measures from the sexual objectification literature, we verified whether greater attention by parents to their children’s body appearance (vs. competence; Study 3) was associated with higher levels of children’s body shame. We also focused on body shame as the main outcome variable in our studies, as it is one of the primary expressions of body image concerns (Gilbert, 2002) that emerges at the early stage of development, leading to a wide range of detrimental consequences for well-being and mental health (Gilbert & Thompson, 2002).

## Body Shame in Childhood

Body shame is conceived as the affective dimension of negative body image and involves adverse feelings that arise when people perceive something wrong related to their body or any part of it (Gilbert, 2002). Moreover, feelings of shame often stem from evaluations of one’s core self as being bad, inadequate, or imperfect, triggered by a sense of personal failure to meet certain standards (e.g., Gilbert, 2002; McKinley & Hyde, 1996).

Far from being harmless, body shame correlates with poorer well-being and mental health, particularly depression, eating disorders, and sexual dysfunction in adults (Augustus-Horvath & Tylka, 2009; Dakanalis et al., 2015; Noll & Fredrickson, 1998; Schaefer et al., 2018; Tiggemann & Williams, 2012; Tylka & Hill, 2004) and adolescents (Iannaccone et al., 2016; Moreira & Canavarro, 2017; Mustapic et al., 2015).

Of relevance to the present research, an increasing number of studies have shown that body shame also emerges among children (Jongenelis et al., 2014), leading to negative consequences. For example, Jongenelis and Pettigrew (2020) found that, for Australian girls and boys aged 6 to 11 years, the experience of body shame was related to body shape concerns and body dissatisfaction. Lindberg and colleagues (2006) examined US girls and boys aged between 10 and 12 years and found negative associations between body shame and body esteem, and positive associations between body shame and past and present dieting behaviors, although these latter relationships were observed only for girls. The consequences of body shame outlined above underscore the need to identify potential contributors of body shame in children.

## The Role of Parental Influence on Children’s Body Image

Parents represent the most important models for children’s development (Maccoby, 1994), and they preeminently influence their children’s attitudes toward body image both directly (e.g., via comments and/or criticism; McCabe & Ricciardelli, 2005) and indirectly (e.g., through the expression of their own negative body image; Czepczor-Bernat et al., 2022; Domoff et al., 2021). For example, Abraczinskas and colleagues (2012) showed that negative body image (i.e., drive for thinness) and bulimic symptomatology in undergraduate women were related to their parents’ weight- and eating-related comments (see also Vincent & McCabe, 2000). Regarding indirect influences, Arroyo and Andersen (2016) found that self-objectification levels in undergraduate women aged 18–25 years were associated with those of their mothers. Similarly, in a multi-generational study, Arroyo and colleagues (2017) observed a significant association between disordered eating attitudes in grandmothers, mothers, and daughters (aged 18–25 years). Despite these relevant findings, when focusing on children’s body shame, research is once again very limited. To the best of our knowledge, the only study investigating the association between parental influence and children’s body shame found that parental body dissatisfaction and perfectionism (mainly from mothers) were positively associated with body shame in children at approximately 13 years of age (Czepczor-Bernat et al., 2022).

Notably, much of this research relies on children’s metaperceptions of their parents’ attitudes (e.g., Lev-Ari & Zohar, 2013) and parents’ self-reported attitudes (e.g., Davison & Birch, 2004; Rogers et al., 2019). However, the findings of these studies have not always been consistent. For instance, a study by Dixon and colleagues (1996) revealed that parents’ encouragement to diet was associated with 8th- and 9th-grade girls’ dieting behaviors when using children’s reports assessing parents’ attitudes. However, no significant relationships were observed between adolescents’ dieting behaviors and parents’ self-reported attitudes toward their children’s dieting (Fulkerson et al., 2002). Considering these discrepancies and to further strengthen the validity of our findings, in our studies we analyzed both children’s and parents’ perspectives. That is, we examined both children’s metaperceptions about parents’ attention to their appearance, in Study 1 and 2, and parents’ self-reported attitudes, in Study 3.

### The Role of Gender in the Intergenerational Transmission of Body Shame

As mentioned above, we hypothesized that the gender of the parent and the child would matter for children’s body shame. Some research has shown (for a review, see Smolak, 2004) that appearance-based messages tend to focus on how women’s bodies look and are more consistent in girls than boys, and girls are more sensitive to these messages than boys. During preadolescence, girls reveal more body concerns than boys (Daniels et al., 2020; Phares et al., 2004) and are more likely to report symptoms of eating disturbance (for a review, see Ricciardelli & McCabe, 2001). Thus, we expected that the association between parents’ attention to their children’s appearance and body shame would be stronger for girls rather than boys.

Most of the existing literature also suggests that mothers, more than fathers, shape their children’s body image attitudes, also during preadolescence (Abramovitz & Birch, 2000; Smolak & Levine, 2001; Wertheim, 2002). For instance, Haines and colleagues (2008) found that mothers’ direct and indirect weight-related behaviors are associated with body dissatisfaction, weight concerns, and dieting behaviors in preadolescents girls and boys. Although the role of fathers has not been as deeply investigated as that of mothers, some research suggests that they also may contribute to their children’s body image attitudes (Agras et al., 2007; Rodgers et al., 2014). Fathers’ dieting behaviors, for example, have been found to influence their daughters’ and sons’ body image concerns (Dixon et al., 1996). Thelen and Cormier (1995) found that, when controlling for children’s BMI, fathers’ but not mothers’ encouragement to diet was significantly related with daughters’ dieting behaviors. In contrast, Smolak and colleagues (1999) found that girls’ body esteem was related to direct maternal influence (i.e., comments about daughters’ weight), but not paternal influence. Overall, it seems that both mothers’ and fathers’ may be relevant to the development of body image concerns in children. In our studies, we attempted to address these issues by comparing the separate role of mothers’ and fathers’ attitudes on children’s body shame.

### Overview of the Present Research

Three studies were conducted to test our hypotheses. Study 1 was designed to provide preliminary evidence of whether children’s metaperceptions of their parents’ attention to their appearance would be associated with higher body shame. Study 2 aimed to replicate and extend the hypothesized pattern of findings by assessing children’s perceptions concerning their own mothers and fathers, respectively. Further, in this study we verified whether the hypothesized effects would remain significant when controlling for other sources of increased body shame outlined by the Tripartite Influence Model (Thompson et al., 1999), that is, peer and media pressure. Data for the first two studies was collected from children aged between 7 and 12 years. To provide stronger validity to our findings, in Study 3 we involved children between 7 and 12 years old and their parents as participants, and tested whether parents’ self-reported attention to their children’s appearance would be associated with increased body shame in children, again, controlling for media and peer influence. Further, in this study, we asked parents to report their children’s BMI, given that it is a crucial individual variable to consider when investigating body image concerns, also affecting parents’ attitudes and behaviors toward their children (Thelen & Cormier, 1995).

In all three studies we verified whether children’s gender would moderate the relationship between parents’ attention to their children’s appearance and body shame. Specifically, based on the literature outlined above, we predicted that the relationship between parents’ attention to their children’s appearance and children’s body shame would be stronger in girls than in boys.

In Study 2 and 3, we also explored the different impact of maternal and paternal attention to their children’s appearance on children’s body shame. Given the mixed findings on the role of mothers and fathers in children’s body image, we did not propose a specific hypothesis but rather posed this test as a research question: *Will the impact of mothers on children’s body shame differ from that of fathers?*

All studies were carried out after obtaining ethical approval from the first author’s university committee, and all measures and procedures have been discussed with education professionals. Data, materials, and supplementary analyses are posted and publicly available on OSF at https://osf.io/vjfu8/?view_only=24bc5e0b8ccb457ba1f7b3aaacec9214.

## Study 1

### Method

#### Participants and Procedure

We recruited a total of 195 children (*n* = 85, 43.60% were female) with a mean age of 8.44 years old (*SD* = 0.62). As our study was conducted in school settings, relevant constraints (e.g., limitations imposed by teachers, the time limit for data collection) did not allow us to determine the sample size a priori. Therefore, we aimed to collect as many participants as possible, depending on the number of participants and classes made available by the primary school local committee. Participants of this study were recruited from a single primary school located in northern Italy. Sensitivity analysis using G*Power (ver. 3.1.9.2; Faul et al., 2007) revealed that our final sample was sufficient to detect a small to medium effect size, *f*^*2*^ = 0.06, assuming an α of 0.05 and a power of 0.80, for a regression including three predictors (i.e., one independent variable, one moderator, and the interaction term).

In the first stage, we obtained approval from the school principal and the class council. Next, we organized a set of meetings to introduce the research project to the parents and education professionals. Then, we sent a letter of introduction to the parents informing them about the aims of the study, the procedure, and the materials. The study was presented as an investigation of children’s perceptions of the importance of physical appearance and body image issues and the invitation included consent forms for parents on behalf of the children. Only children who received parental consent and provided assent forms were recruited for the study. Participants individually completed a survey during regular class time with either the lead author or research assistants who read the instructions for the task. Upon completion of the task, participants were thanked and invited to ask any questions about the survey that they had completed.

### Measures

The measures included in the survey are presented below. Unless otherwise specified, all items were scored on a 5-point scale ranging from 1 (*absolutely not*) to 5 (*absolutely yes*), and 3 representing a neutral score (*maybe not, maybe yes*).

#### Children’s Metaperceptions of Parents’ Attention to their Appearance

Children’s metaperceptions were assessed with four items adapted from previous research (e.g., McKinley & Hyde, 1996) and tailored for a child sample. We reworded items of the Surveillance subscale of the Objectified Body Consciousness Scale for Youth (OBC-Y; Lindberg et al., 2006) and asked children to indicate their level of agreement. The following items were used: “My parents often compare how I look with how other people look”, “During the day, my parents think about how I look many times”, “My parents often worry about whether the clothes I am wearing make me look good”, and “My parents often worry about how I look to other people” (α = 0.61). Items’ scores were averaged to establish an index of perceived parents’ influence, with higher scores reflecting greater children’s metaperceived parental attention to their appearance.

#### Children’s Body Shame

Body shame was assessed with items from the Body Shame subscale of the OBC-Y (Lindberg et al., 2006) which captures feelings of shame due to the body appearance. The subscale comprises five items (e.g., “I would be ashamed for people to know what I really weigh”; α = 0.68) the scores of which were merged to form a composite index of body shame, with higher scores denoting greater feelings of shame toward the body.

## Results and Discussion

There was no significant difference between girls’ and boys’ metaperceptions of parents’ attention to their appearance (*M* = 2.47, *SD* = 1.01) nor for children’s body shame (*M* = 2.35, *SD* = 1.00), all *t’*s < 1.618, and *p’*s > .107. A positive correlation emerged between metaperceptions of parents’ attention to children’s appearance and body shame in children, *r* = .38, *p* < .001, suggesting that the more children perceived their parents giving attention to their appearance, the higher their feelings of body shame.

To test the hypotheses that children’s metaperceptions about parents’ attention to their appearance were related to children’s body shame and that the relationship would be moderated by children’s gender, we used the PROCESS Macro for SPSS (Hayes, 2013; Model 1). Specifically, we entered children’s metaperceptions as the independent variable, children’s gender as the moderator, and body shame as the dependent variable. The model explained approximately 15% of the variance in children’s body shame. Findings confirmed the hypothesized relationships, showing that children’s metaperceptions of their parents’ attention to their appearance were positively related to their levels of body shame, *B* = 0.42, *SE =* 0.09, *p* < .001. However, children’s gender did not moderate this association, *B* = − 0.11, *SE =* 0.13, *p* = .419.

Findings from Study 1 provided preliminary evidence for the link between children’s metaperceived parents’ attention to their appearance and children’s body shame: perceiving parents’ attention to their bodies was related to higher levels of body shame in children. Contrary to our expectations, this pattern of findings did not differ depending on children’s gender, indicating that parents’ attention to children’s appearance, at least when self-perceived by children, affects girls and boys to the same extent.

### Study 2

Study 2 was designed to extend the findings of the previous study in two main directions. In contrast to Study 1, this study focused on the distinct roles of mothers and fathers. Thus, we assessed the association between children’s metaperceived mothers’ and fathers’ attention to their appearance and body shame in girls and boys. Furthermore, we verified the link between these children’s metaperceptions and their body shame when controlling for the influence of peer and media, the two other sources proposed by the Tripartite Influence Model (Thompson et al., 1999). Finally, as in Study 1, we examined whether children’s gender moderated the tested relationships.

### Method

#### Participants and Procedure

We recruited a total of 163 participants (*n* = 79, 48.47% were female) with a mean age of 9.35 (*SD* = 0.96) from a primary school located in northern Italy. We planned to follow the same procedure as Study 1. However, due to the COVID-19 pandemic, in-person data collection was interrupted after 47 participants (*n* = 19, 40.43% were female). We converted the paper-based survey to an online survey platform and the remaining 125 participants (*n* = 62, 49.6% were female) completed the survey online. To ensure that children were able to understand the online response format, they were invited to correctly answer a question based on a short story that we created (see the online supplemental material on OSF: https://osf.io/vjfu8/?view_only=24bc5e0b8ccb457ba1f7b3aaacec9214) using a scale ranging from 1 (*strongly disagree*) to 5 (*strongly agree*). Only children who provided the correct answer (*n* = 116, 92.8%) were included in our analyses. Considering the effects due to different data collection methods (De-Leeuw & Hox, 2018), we first performed all our analyses controlling for the method type (in-person vs. online). Data collection condition did not explain a significant amount of variance in any case, and the results did not differ from those obtained from analyses in which data condition was not included. Accordingly, the analyses presented below do not include method type as a covariate.

### Measures

Measures included in the survey are presented below. Unless otherwise specified, all items were scored on a 5-point scale ranging from 1 (*absolutely not*) to 5 (*absolutely yes*), and 3 representing a neutral score (*maybe not, maybe yes*).

#### Children’s Metaperceptions About Parents’ Attention to Children’s Appearance

Children’s metaperceptions were assessed through two adapted versions of the OBC-Y used in Study 1, with reworded items to capture perceptions of their appearance being monitored by their mothers and fathers, respectively. Children responded to eight items assessing their metaperceptions about their mothers’ (four items) and fathers’ (four items) attention to their appearance: “My mother/father compares how I look with how other people look”, “During the day, my mother/father think about how I look many times”, “My mother/father often worry about whether the clothes I am wearing make me look good”, and “My mother/father often worry about how I look to other people” (α = 0.69 and α = 0.74 for children’s metaperceptions about mothers’ and fathers’ attention to children’s appearance, respectively). For both scales, responses to items were averaged to create separate scores for metaperceived attention to children’s appearance by mothers and fathers, with higher scores denoting greater metaperceived mothers’ and fathers’ attention.

#### Children’s Body Shame

As in Study 1, body shame was measured with the five items (α = 0.85) from the Body Shame subscale of the OBC-Y (Lindberg et al., 2006).

#### Peer Influence

Peer influence was assessed with three items selected from the Likability subscales of the I-PIEC (Oliver & Thelen, 1996). The subscale measures the degree to which children believe that changes in their body image will increase their likability with peers. We adapted these items to assess peer pressure regarding the thin ideal for girls and the muscular ideal for boys (Jones & Crawford, 2005; Ricciardelli & McCabe, 2001). Specifically, the following items were used: “If I were thinner/more muscular, I think that children would want to sit next to me more often”, “I think that children think I would look better thinner/more muscular”, “I think that children would talk to me more if I were thinner/more muscular” (α = 0.87). Mean scores were calculated to estimate peer influence, with higher scores denoting greater perceptions of likability by peers if thinner/more muscular.

#### Media Influence

To assess media influence, we used the Internalization subscale of the Multidimensional Media Influence Scale (Harrison, 2009). The subscale comprises six items that assess the internalization of beauty ideals in the media as one’s own personal beauty standard (e.g., “I try to look like the actors or actresses in movies”; α = 0.84). According to few teachers whose classes participated in the research project, one item of the subscale (i.e., “I try to look like the models in magazines”) was not applicable to our sample, hence, it was excluded. The final subscale administered to participants comprised five items. Items’ scores were averaged to form an index of media influence, with higher scores reflecting participants’ greater perceptions of media pressure.

## Results and Discussion

Sensitivity analysis showed that our final sample was sufficient to detect a small to medium effect size, *f*^*2*^ = 0.09, assuming an α of 0.05 and a power of 0.80, for a regression analysis with 6 predictors (two independent variables, one moderator, one interaction terms, and two covariates). Descriptive statistics and correlations for the study variables are presented in Table 1.

**Table 1:** Descriptive Statistics and Correlations for Study 2 and 3 Variables

Variable			1	2	3	4	5	6	7	8
1. Mothers’ attention to children’s appearance			–	.52^***^	.29^***^	.21^***^	.41^***^	.13	–	− .11
2. Fathers’ attention to children’s appearance			.46^***^	–	.39^***^	.29^***^	.33^***^	.11	–	− .08
3. Children’s body shame			≈ .00	.22	–	.56^***^	.56^***^	.09	–	− .01
4. Peer influence			− .02	− .05	.52^***^	–	.48^***^	− .17^*^	–	.03
5. Media influence			.13	.13	.49^***^	.55^***^	–	.29^***^	–	.11
6. Children’s gender (0 = boys, 1 = girls)			− .04	− .15	− .16	− .24^*^	− .01	–	–	.10
7. Children’s BMI			− .02	.12	− .03	− .10	− .06	− .03	–	–
8. Data collection method (0 = in person, 1 = online)			.02	.33^**^	.20	.12	.27^*^	.05	.12	–
Mean (*SD*)	*Study 2*	Boys (*N* = 84)	2.17 (0.81)	1.78 (0.78)	2.10 (1.04)	1.85 (1.07)	**2.10 (1.00)**	–	–	–
		Girls (*N* = 79)	2.40 (0.96)	1.96 (0.90)	2.28 (1.01)	1.51 (0.88)	**2.71 (1.05)**	–	–	–
	*Study 3*	Boys (*N* = 26)	-12.46 (14.26)	-5.46 (14.07)	2.14 (0.98)	**1.61 (0.78)**	1.82 (0.89)	–	17.89 (4.12)	–
		Girls (*N* = 44)	-13.50 (11.21)	-9.75 (14.51)	1.86 (0.72)	**1.27 (0.61)**	1.81 (0.84)	–	17.70 (3.03)	–

*Note*. Study 2 *N* = 163 and Study 3 *N* = 70 parents-child triads. Study 2 correlations are indicated above the diagonal, Study 3 correlations are reported below the diagonal. Means reported boldface indicate significant mean gender differences. Study 2 assessed metaperceived parents’ attention to children’s appearance. Study 3 assessed parents’ self-reported attention to children’s appearance. ^*^p < .05. ^**^p < .01. ^***^p < .001.

To test our hypotheses, we ran two moderation analyses using the PROCESS Macro for SPSS (Hayes, 2013; Model 1). Children’s metaperceptions of their mothers’ and fathers’ attention to their appearance were entered as the independent variables, children’s gender was the moderator, and their level of body shame was the outcome variable. In addition, the interaction between the moderator and the independent variable was included in the model. Finally, peer and media influence were considered as covariates.

Results revealed that children’s metaperceptions of their fathers’ (but not mothers’) attention to their appearance was positively related to children’s body shame (see Table 2). For the covariates, both peer influence and media influence were related to higher body shame in children. Consistent with Study 1, the gender of the child did not moderate the association between children’s metaperceptions of mothers’ or fathers’ attention to children’s appearance and body shame.

**Table 2:** Summary of Results for Moderation Analyses for Study 2 and 3

Predictors		Dependent Variable Body Shame			
		Study 2 (*N* = 163)		Study 3 (*N* = 70)	
		Mothers × Children’s Gender	Fathers × Children’s Gender	Mothers × Children’s Gender	Fathers × Children’s Gender
		*B (SE)*, 95% CI [LL, UL]	*B (SE)*, 95% CI [LL, UL]	*B (SE)*, 95% CI [LL, UL]	*B (SE)*, 95% CI [LL, UL]
Mothers’ attention to children’s appearance	(a)	.01 (.12) [-0.2270, 0.2405]	− .02 (.09) [-0.1889, 0.1454]	− .01 (.01) [-0.0272, 0.0142]	− .01 (.01) [-0.0261, 0.0045]
Fathers’ attention to children’s appearance		.22 (.09)^*^ [0.0506, 0.3953]	.22 (.09)^*^ [0.0478, 0.3981]	.02 (.01)^*^ [0.0021, 0.0310]	.02 (.01)^*^ [0.0020, 0.0305]
Children’s gender (0 = boys, 1 = girls)	(b)	.18 (.34) [-0.4944, 0.8604]	.07 (.14) [-0.1976, 0.3430]	− .06 (.19) [-0.4276, 0.3076]	− .04 (.18) [-0.4037, 0.3171]
Interaction (a × b)		− .05 (.14) [-0.3362, 0.2345]	.02 (.15) [-0.2764, 0.3074]	− .01 (.01) [-0.0351, 0.0204]	− .02 (.01) [-0.0447, 0.0040]
Peer influence		.36 (.08)^***^ [0.2068, 0.5160]	.37 (.08)^***^ [0.2171, 0.5195]	.45 (.16)^**^ [0.1433, 0.7637]	.43 (.15)^**^ [0.1295, 0.7327]
Media influence		.32 (.08)^***^ [0.1691, 0.4752]	.32 (.08)^***^ [0.1662, 0.4680]	.25 (.13) [-0.0043, 0.4984]	.26 (.12) [-0.0672, 0.5784]
Child BMI		–	–	.00 (.02) [-0.0524, 0.0463]	.00 (.02) [-0.0476, 0.0495]
Data collection method		–	–	.00 (.20) [-0.4089, 0.4067]	− .02 (.20) [-0.4282, 0.3729]
*R* ^2^		.45	.45	.39	.41
*f* ^2^		.82	.82	.64	.69
*F*		21.42^***^	21.38***	4.84^***^	5.34^***^
*df*		(6, 156)	(6, 156)	(8, 61)	(8, 61)

*Note.* Study 2 *N* = 163 and Study 3 *N* = 70 parents-child triads. Study 2 assessed metaperceptions of parents’ attention to children’s appearance. Study 3 assessed parents’ self-reported attention to children’s appearance.

^*^p < .05. ^**^p < .01. ^***^p < .001.

Taken together, these results replicated and integrated the findings of Study 1. There was a positive correlation between children’s metaperceptions of parents’ attention to their appearance and children’s body shame. However, when children’s metaperceptions of mothers’ and fathers’ attention to children’s appearance were considered together in the regression analysis, only metaperceptions of fathers’ attention remained significant. This result suggests that the attention given by fathers to children’s appearance may be more strongly associated with children’s body shame than the attention given by mothers. Notably, the link between metaperceptions of fathers’ attention to their appearance and children’s body shame remained significant when controlling for peer and media influence. Second, unlike our initial hypotheses but confirming the pattern of results in Study 1, child gender did not moderate any of the tested effects, suggesting once again that metaperceptions of parents’ attention to children’s appearance is not gender-specific, but rather has a similar impact on girls and boys.

### Study 3

Study 3 aimed to enhance the validity of our previous findings by considering parents’ self-reported attention to children’s appearance, rather than children’s metaperceptions. More specifically, in Study 3 we gathered data from both children and their parents and investigated the link between mothers’ and fathers’ self-reported attention to their children’s appearance and body shame in girls and boys. This relationship was examined while considering the influence of peers and media, and children’s BMI. As in Study 1 and 2, we tested the moderating effect of children’s gender, anticipating stronger associations among girls compared to boys.

### Method

#### Participants and Procedure

A total of 103 children, 99 mothers, and 78 fathers agreed to participate in the study. Given the purpose of the study, we only analyzed data from complete parent-child triads. Accordingly, our final sample comprised 70 parent-child triads (*N* = 44 children, 62.86% were female) recruited from a single primary school located in northern Italy. Children were between the ages of 7 and 12 years old (*M* = 9.61, *SD* = 0.86). Demographic information for parents can be found in the online supplemental material on OSF: https://osf.io/vjfu8/?view_only=24bc5e0b8ccb457ba1f7b3aaacec9214.

As in Study 2, in-person data collection was interrupted after 46 triads due to the COVID-19 pandemic. We continued recruiting triads online by converting the paper-based survey to an online survey platform. To ensure that children were able to understand the response format, they were invited to correctly answer the question based on the same short story we created for Study 2 ranging from 1 (*strongly disagree*) to 5 (*strongly agree*). All the children provided the correct answer and were included in the analyses. As data collection method affects some of the relationships between our variables, the analyses presented below include data collection method as a covariate.

### Measures

#### Parents’ Measures

##### Parents’ Attention to Children’s Appearance

We used an adapted version of the Self-Objectification Questionnaire (SOQ; Fredrickson et al., 1998; Strelan & Hargreaves, 2005) to assess the degree to which parents valued their child’s external appearance more than their non-observable qualities. Specifically, parents ranked the importance of 10 body attributes for their child’s physical self-concept, with five attributes focused on external appearance (i.e., *“firm/sculpted muscles, height, measures, physical attractiveness, weight”*) and five attributes focused on non-observable competence-based attributes (i.e., *“energy level*, *health*, *physical coordination*, *physical fitness level*, *physical strength”*). One of the original appearance-based attributes, *sex appeal*, was replaced with *height*, which was more appropriate for the evaluation of children (see Jongenelis et al., 2014 for a similar procedure). Participants ranked the importance of these attributes from 1 (*least important*) to 10 (*most important*). Instructions given to parents were the following: “We ask you now to think about how you perceive your son/daughter. Listed below are 10 different attributes that you can value as more or less important. When you think about your son/daughter, which of these attributes do you value most? Please rank the following features from the most (10) to the least important (1) when thinking about your son/daughter.” The final score for each participant was then computed by subtracting the sum of the ranks for the non-observable competence-based attributes from the sum of the ranks for the observable appearance-based attributes. Scores ranged from − 25 to + 25, with higher scores reflecting greater emphasis on appearance-based attributes.

#### Body Mass Index

Parents were also asked to report their children’s weight (in *kg*) and height (in *cm*) to compute the child’s BMI score.

#### Children’s Measures

The same measures from the previous studies were used to assess children’s body shame (α = 0.67), peer influence (α = 0.73), and media influence (α = 0.79).

## Results and Discussion

Sensitivity analysis showed that our final sample was sufficient to detect a medium to large effect size, *f*^*2*^ = 0.24, assuming an α of 0.05 and a power of 0.80, for a regression including 8 predictors (two independent variables, one moderator, the interaction term, four covariates). Descriptive statistics and correlations for the study variables are presented in Table 1.

To test the link between mothers’ and fathers’ self-reported attention to children’s appearance with children’s body shame and investigate whether this link varied as a function of child gender, we ran two moderation analyses similar to the one conducted in Study 2. Mothers’ and fathers’ attention to children’s appearance were entered as the independent variables in the model, children’s gender was the moderator, and the dependent variable was children’s body shame, as well as the interaction term. We performed our analysis controlling for peer and media influence, children’s BMI, and the method of data collection. As presented in Table 2, mothers’ attention to children’s appearance was not associated with children’s body shame. However, there was a small effect for fathers’ attention to children’s appearance on children’s body shame, whereas child gender did not moderate these associations. Both peer influence and media influence were positively related to children’s body shame.

Overall, the findings of Study 3 expanded on the results of the previous studies by considering the impact of parents’ self-reported perceptions of children’s appearance attributes (vs. children’s metaperceptions). Once again, we found that fathers’, but not mothers’, attention to children’s appearance was related to higher body shame in girls and boys. This relationship was significant when controlling for peer and media influence, children’s BMI, and data collection method. Further, we replicated findings from the prior studies by showing that these patterns did not differ between girls and boys.

### General Discussion

Experiencing body shame is painful for individuals, and it often begins at a very young age. Therefore, exploring potential factors associated with this experience during childhood is crucial, especially when considering its short-term and long-term consequences. By adopting an integrated view of the Tripartite Influence Model (Thompson et al., 1999) and Objectification Theory (Fredrickson & Roberts, 1997), across three studies we investigated the associations between parents’ attention to their children’s appearance (a construct that we derived from the sexual objectification literature) and girls’ and boys’ body shame. The results of these studies provide support for a reliable link between parents’ attention to children’s appearance, both perceived by children and self-reported by parents, and children’s body shame. Importantly, this link remained significant also when controlling for the two other main sources of body image concerns identified by the Tripartite Influence Model, i.e., peers and media influence.

Interestingly and contrary to our hypothesis, we did not find evidence that the gender of the child moderated the link between parents’ attention to their appearance and body shame. This result was consistent across all three studies. Although past research has revealed that girls may be more vulnerable to appearance messages than boys (Ricciardelli & McCabe, 2001), our data suggest that parental attention to children’s appearance may be related to greater body shame in children, regardless of the child’s gender. One possibility for this pattern may be the developmental age of the children in our studies who would be in the very early stages of puberty or not yet entered puberty, and therefore the focus on physical changes in bodily appearance is not as salient (Ricciardelli & McCabe, 2001). It is noteworthy that some previous research examining antecedents of body image concerns in children of a similar age to our studies also did not find the expected gender differences (see, e.g., Jongenelis & Pettigrew, 2020).

Perhaps even more importantly, we found that when considering fathers’ and mothers’ attention to their children’s appearance together, fathers’ perceptions were more strongly related to children’s body shame than mothers’ perceptions. This pattern of findings occurred both when considering children’s metaperceptions (Study 2) and parents’ self-reported views (Study 3). Even though this result may seem in contrast with some prior literature suggesting that, during development, mothers more than fathers influence their children’s body image attitudes (Smolak & Levine, 2001), it is important to note that most of the research so far has focused on children’s body dissatisfaction (e.g., Dahill et al., 2021; Solano-Pinto et al., 2021). Body shame and body dissatisfaction, although related, are distinct constructs. Specifically, body dissatisfaction refers to the discontent with one’s overall physical attractiveness or body parts (Cash et al., 2004), while body shame describes the experience of being embarrassed by the body, and wanting to hide one’s body from others, because it does not meet the cultural standards of beauty, along with negative feelings toward the self in general (McKinley & Hyde, 1996).

In addition, most research in this area has focused on the mother-child relationship, while the role of fathers remains understudied. Thus, our results contribute to understanding the relevant role played by fathers in shaping their children’s body image concerns and, specifically, their body shame. A possible explanation for this crucial finding builds on Objectification Theory, which claims that sociocultural messages stressing the importance of the body and physical appearance rely on a male point of view. Therefore, fathers’ attitudes toward their children’s physical appearance may be more powerful than those of their mothers, especially when considering body shame - the result of failing to meet internalized cultural body standards - as the outcome. Further, given the age of the children in our studies, it is possible that, in younger individuals, fathers are more influential in affecting children’s body shame, while the role of mothers may become more prominent during early adolescence.

Another interesting result from our studies concerns the comparison between children’s metaperceptions of parents’ attention to children’s appearance and mothers’ and fathers’ self-reported attention to children’s appearance. Our findings revealed that the association between fathers’ attention to children’s appearance and body shame was consistent with children’s metaperceptions of fathers attention to their appearance, whereas this was not observed for mothers. In fact, in Study 2 metaperceptions of mothers’ attention to their children’s appearance was related to children’s body shame, whereas in Study 3 mothers’ self-reported attention to their children’s appearance was not related to children’s body shame. These differences may be because mothers shape children’s body image attitudes more subtly than fathers, for example through being dissatisfied with their own bodies rather than directly expressing concerns for their children’s appearance (Arroyo & Andersen, 2016). It may also be that in our sample mothers may have been more sensitive to social desirability and thus may have adjusted their responses more than fathers. In fact, although we did not directly assess social desirability, some prior work reveals gender effects when investigating this bias (e.g., Chung & Monroe, 2003), with women displaying higher social desirability than men. This would help to explain the different results obtained when children’s metaperceptions vs. parents’ self-reported perceptions were considered. However, further research is required before any explanation for this result can be made. Overall, the converging evidence when considering both children’s (Study 1 and 2) and fathers’ (Study 3) perspectives increased confidence in the observed patterns, partially resolving the discrepancies in previous research that considered either children’s metaperceptions or self- parents’ self-reported attitudes.

Furthermore, it is important to consider the measures adapted from the sexual objectification literature that we used to assess parents’ attention to appearance in our studies. In Study 1 and 2, the measure used to assess children’s metaperceptions of parents’ attention to their appearance focused on the degree to which children perceived their parents monitoring their child’s physical appearance by, for instance, comparing their child’s body shape to those of other children. In Study 3, the measure completed by parents assessed the value that both mothers and fathers placed on their children’s physical appearance compared to their physical competence. Thus, our findings indicate that body shame in children is associated with both children’s perceptions of their appearance being monitored by their mothers and fathers and the emphasis that their parents place on their appearance, when compared to other qualities.

Finally, though the focus of the current research was on the role of parents, our studies also provided further evidence on the importance of peers and media in eliciting children’s body shame. In fact, it is noteworthy that in Study 2 and 3 both peers and media demonstrated stronger associations with children’s body shame than parental attention to children’s appearance. These results may be attributed to the age range of our participants (7 to 12 years old). Indeed, during the process of growing up, children tend to allocate more time to interacting with peers rather than engaging with parents (Berndt, 1996; Larson & Richards, 1991), and their exposure to and interaction with media also increases (see Daniels et al., 2020), suggesting that, at least at this stage of development, the role of peers and media may surpass that of parents in contributing to children’s body shame.

### Limitations and Future Research Directions

Despite the relevance of our findings, we note some limitations that could guide future research. Firstly, our study is based on correlational data, thereby preventing any conclusion regarding the causal relationship between the variables investigated. Consequently, we cannot assume that parental attention to children’s appearance leads to body shame in girls and boys, as it is equally possible that children with higher levels of body shame influence the extent to which their parents focus on their appearance and value their bodies. Therefore, future experimental and longitudinal research is required. For instance, scholars could investigate how body shame develops over time and how the level of parental influence changes at different stages of development. It would be particularly valuable to explore this phenomenon during the transition from childhood to adolescence, as this critical period is characterized by profound changes happening at both the psychological (e.g., social and emotional changes) and physical (e.g., physical growth and changes to children’s sexual organs) level, that are particularly relevant to girls’ and boys’ psychological well-being and health (Ricciardelli & McCabe, 2001).

A further limitation is the “borderline” reliability (Kline, 1986; Ponterotto & Ruckdeschel, 2007) of some of our measures, especially the ones assessing parents’ attention to children’s appearance (α = 0.61) and body shame (α = 0.68) in Study 1, maternal attention to children’s appearance in Study 2 (α = 0.69), and body shame (α = 0.67) in Study 3. Although different steps have been implemented before data collection (i.e., teachers verified the understandability of the items; trained researchers were present during data collection; scales responses were reduced from 7 to 5 steps), some alphas were still relatively low. The low alphas observed may be attributed to the limited number of items employed to measure the constructs and the age range of the participants included in our research. However, the low reliability was specific to only a few measures, while the remaining alphas ranged from acceptable to good.

It is worth mentioning that, in Study 3, we measured parents’ self-reported attention to their children’s appearance without also assessing children’s metaperceptions, as we did in Study 1 and 2, which prevented us from examining the convergence between these perceptions. Future research should consider measuring both children’s metaperceptions of their parents’ attention to their appearance and parents’ self-reported attention within the same study. This approach would provide insight into how children’s metaperceptions about their parents’ attitudes align with their parent’s self-reported attitudes and which variable has a greater impact on children’s body shame.

As previously acknowledged in the introduction, our research has primarily focused on the role of parents in shaping girls’ and boys’ body shame while also considering the influence of peers, media, and individual factors such as the child’s BMI. However, it is important to recognize that there are several other dispositional variables and social experiences that could potentially work alongside the sociocultural agents considered in our studies. For example, body image concerns of parents themselves (Arroyo & Andersen, 2016) or attributions made by peers regarding the significance of body weight and shape for popularity (Bigler et al., 2019; Matera et al., 2013) have also been found to have an influence on children’s negative body image. Future research could explore the interactions between these variables and parental influence, and how early these agents emerge in the lives of children, to gain a better understanding of the dynamics at play in shaping children’s body shame.

#### Practice Implications

Children’s social environments play a crucial role in shaping their body image, whether positively or negatively. Our findings suggest that the attention parents give to their children’s appearance is associated with more body shame in young girls and boys. Interestingly, this attention to appearance does not stem from explicit compliments or criticism, which have been previously linked to various body image concerns in children (Keery et al., 2004; Rodgers et al., 2020). Instead, it is conceived as the attention parents pay to their children’s appearance (Study 1 and 2) and the emphasis they place on their bodily features compared to other qualities (Study 3). Thus, mere attention to the physical appearance of children may communicate the value of their physical appearance to them in a way that is detrimental, and thus deemphasizing appearance in everyday life may be protective against negative body image.

Professionals can collaborate with parents of young girls and boys to explore alternative ways of interacting with their children and engaging in conversations that prioritize qualities beyond physical appearance. For instance, emphasizing the importance of personality, emotional expression, and emotional regulation rather than focusing merely on body measurements and appearance can help children understand that their self-worth is not contingent only on their physical appearance. Thus, interventions designed to reduce negative body image in young girls and boys should offer parents alternative communication strategies to adopt when interacting with their children.

## Conclusion

In the present research, we adopted an integrated view of the Tripartite Influence Model and Objectification Theory to explore potential correlates of children’s body shame. Overall, this research provides evidence for the association between parents’ attention to their children’s appearance and children’s body shame beyond peer and media influence that did not differ between boys and girls. Given the pervasiveness of body image concerns in childhood, preventing the development of negative attitudes toward the body is of utmost importance. These findings underscore the importance for parents to deemphasize appearance in young children, especially fathers, as children appear to be attuned to their parents’ attention to their appearance even when not explicitly critical which may attenuate the link to body shame in those children. Future research should continue to examine the nuanced and less visible ways in which attention to appearance is harmful for children’s body image.

## Data Availability

The data and materials used in the research are available. The data and materials used in the research can be obtained at https://osf.io/vjfu8/?view_only=24bc5e0b8ccb457ba1f7b3aaacec9214 or by emailing luca.andrighetto@unige.it
